# Perspectives on the essence and drivers of aging

**DOI:** 10.1093/lifemedi/lnaf006

**Published:** 2025-02-22

**Authors:** Haim Y Cohen, Vera Gorbunova, Steve Horvath, Brian K Kennedy, Wei Li, João Pedro de Magalhães, Andrei Seluanov, Moshi Song, Keiichiro Suzuki

**Affiliations:** The Mina and Everard Goodman Faculty of Life Sciences, Bar-Ilan University, Ramat-Gan 52900, Israel; Department of Biology, University of Rochester, Rochester, NY 14627, United States; Department of Medicine, University of Rochester Medical Center, Rochester, NY 14627, United States; Human Genetics, David Geffen School of Medicine, University of California Los Angeles, Los Angeles, CA 90095, United States; Healthy Longevity Translational Research Programme, Yong Loo Lin School of Medicine, National University of Singapore, Singapore 117597, Singapore; State Key Laboratory of Organ Regeneration and Reconstruction, Institute of Zoology, Chinese Academy of Sciences, Beijing 100101, China; Beijing Institute for Stem Cell and Regenerative Medicine, Beijing 100101, China; Genomics of Ageing and Rejuvenation Lab, Institute of Inflammation and Ageing, University of Birmingham, Birmingham B15 2TT, United Kingdom; Department of Biology, University of Rochester, Rochester, NY 14627, United States; Department of Medicine, University of Rochester Medical Center, Rochester, NY 14627, United States; State Key Laboratory of Organ Regeneration and Reconstruction, Institute of Zoology, Chinese Academy of Sciences, Beijing 100101, China; Beijing Institute for Stem Cell and Regenerative Medicine, Beijing 100101, China; Institute for Advanced Co-Creation Studies, Osaka University, Osaka 560-8531, Japan; Graduate School of Engineering Science, Osaka University, Osaka 560-8531, Japan; Graduate School of Frontier Bioscience, Osaka University, Osaka 565-0871, Japan

Aging is commonly associated with the decline in the function of tissues and organs, along with increased prevalence of age-related diseases. At the “International Symposium on Aging and Rejuvenation” hosted by the Institute of Zoology, Chinese Academy of Sciences, a panel of experts convened to delve into critical topics, including the biomarkers indicative of aging and the factors that accelerate this process. Throughout the symposium, each expert presented their research findings and offered profound insights into the mechanisms, biomarkers, and potential interventions for combating aging. Here, several of the editorial board members at the symposium answer the questions posed by *Life Medicine*.

## What is the essence of aging? What drives the aging process?


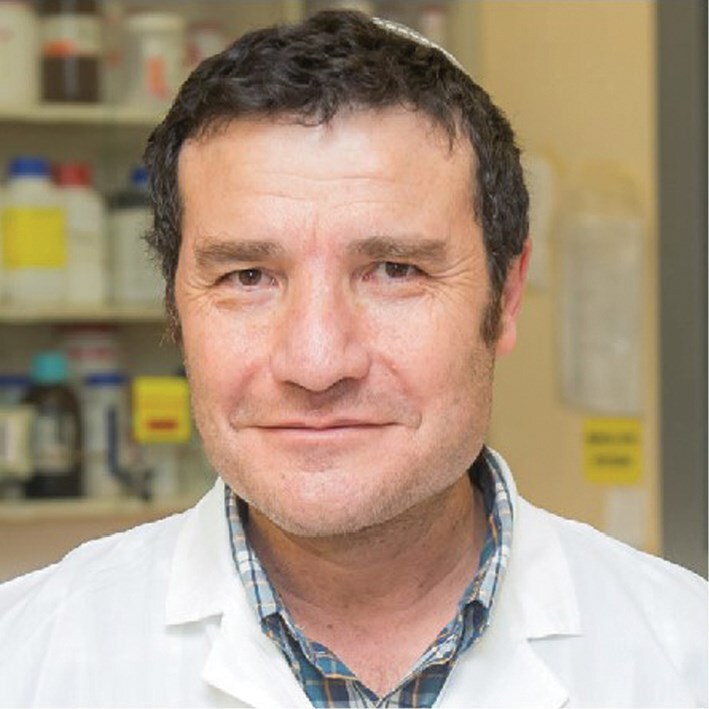


**Haim Y. Cohen**

There are several drivers of aging, making it challenging to pinpoint any single one. Among them, inflammation stands out. It accompanies us throughout life and can be considered a chronic driver of aging. Fortunately, we have made progress in understanding how this phenomenon develops and identifying the key regulators underlying it. This knowledge may enable us to find drugs that target these processes, potentially extending longevity.


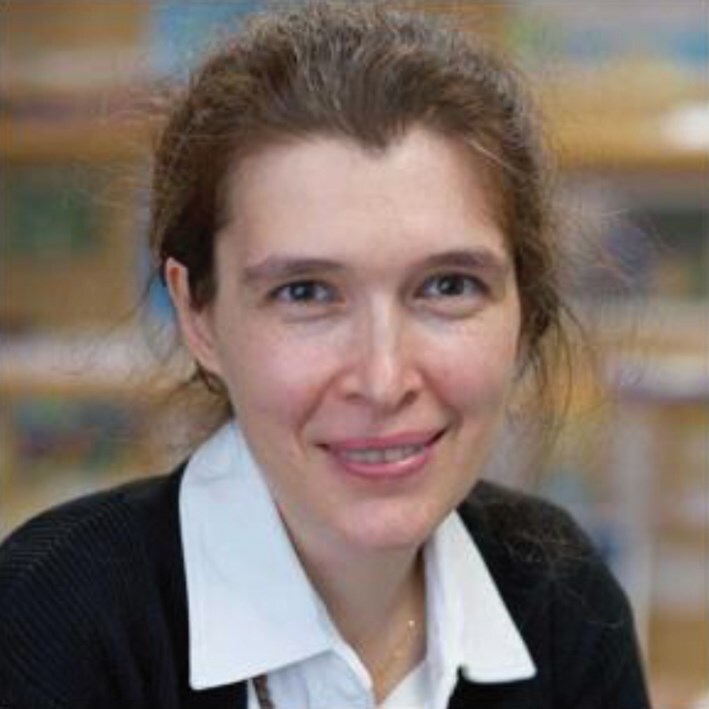


**Vera Gorbunova**

Aging is the process of gradual and generalized deterioration that occurs over lifetime of an organism. It includes damage to DNA and proteins and deterioration of the epigenetic structure of chromatin.


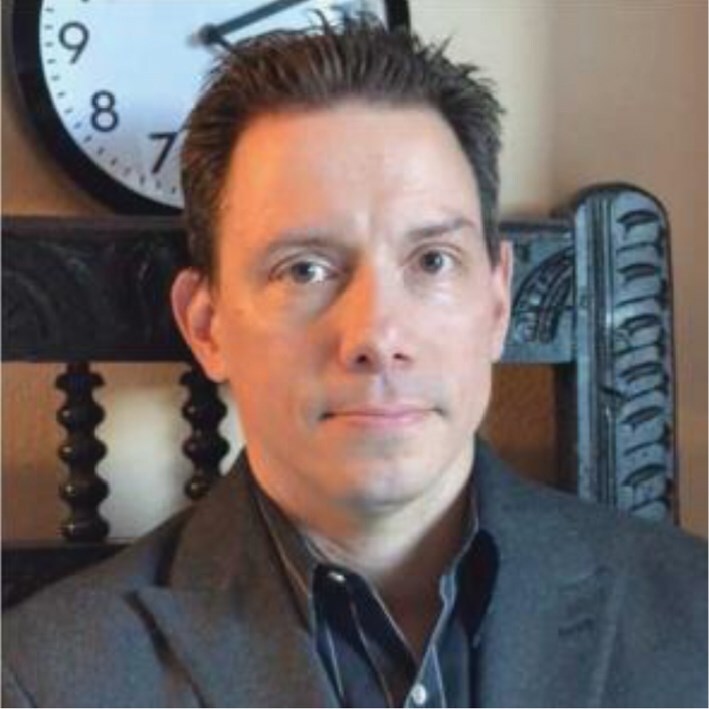


**Steve Horvath**

Epigenetic changes play a significant role. Age-related epigenetic modifications, including DNA methylation and histone modifications, impair the cell’s ability to respond appropriately to internal and external signals, leading to disrupted communication both within individual cells and across different tissues.


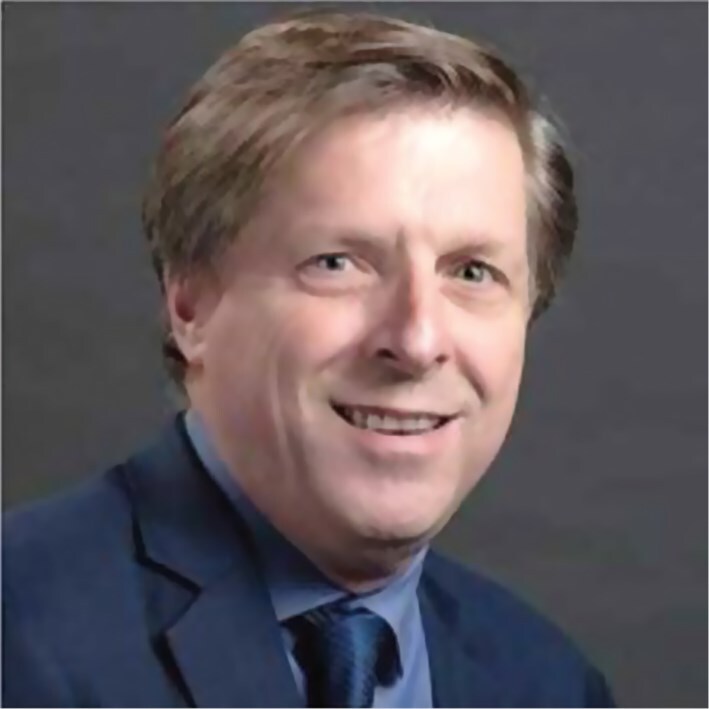


**Brian K. Kennedy**

I think of aging as a loss of homeostasis that results in susceptibility to any of a range of chronic diseases depending on a person’s genetics, lifestyle, environment, etc. Our bodies are designed to keep us functional, but events happen as we age, including many forms of damage, stochastic changes, and external challenges (e.g. infections), and while the network compensates, it slowly gets degraded. Hallmarks and pillars of aging are components or readouts of network function. Therefore, the interventions that extend healthspan and lifespan are targeting nodes in the network (e.g. mTOR) or rejuvenating its vital components (e.g. adult stem cells). Aging, in my view, is caused by a constellation of events, most of which on their own having a small impact, but ultimately triggering system dysfunction.


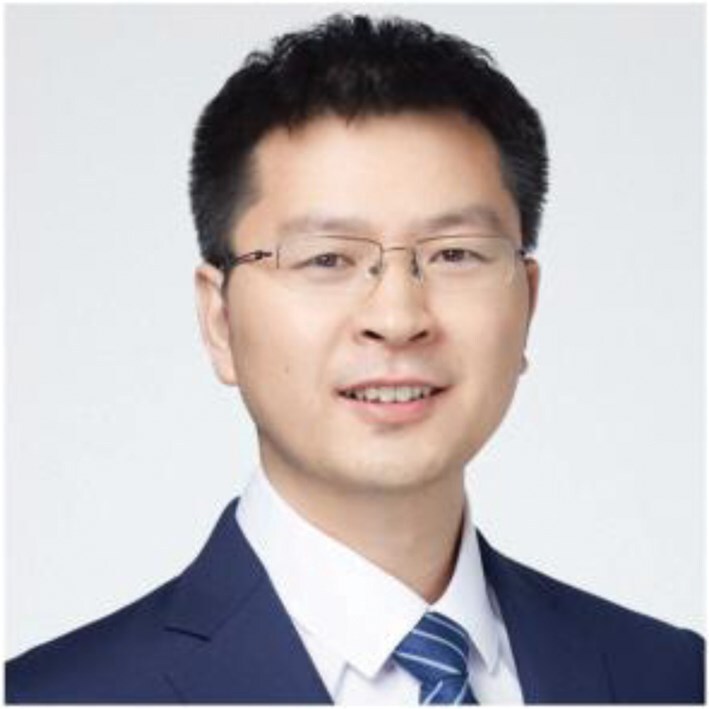


**Wei Li**

If we consider the human body as a complex machine assembled from many molecular machines, the essence of aging is the inevitable damage to the molecular machines being running for a long time, which in turn triggers a series of disordered physiological cascade reactions, leading to abnormal biological functions. From this perspective, better repair ability for molecular machine damage or timely termination of abnormal physiological cascade reactions may delay aging.


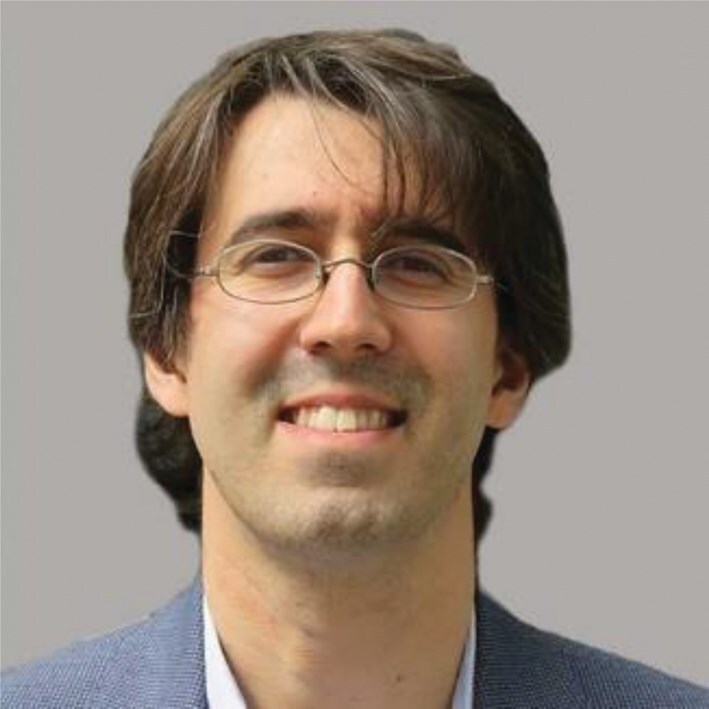


**João Pedro de Magalhães**

We do not yet know the drivers of human aging. There are several hypotheses, such as DNA damage, epigenetic changes, and telomere shortening, but none have been proven empirically. It is also possible that aging is not driven by random molecular damage but rather by programmatic processes that start during development and run-on into adulthood and become detrimental.


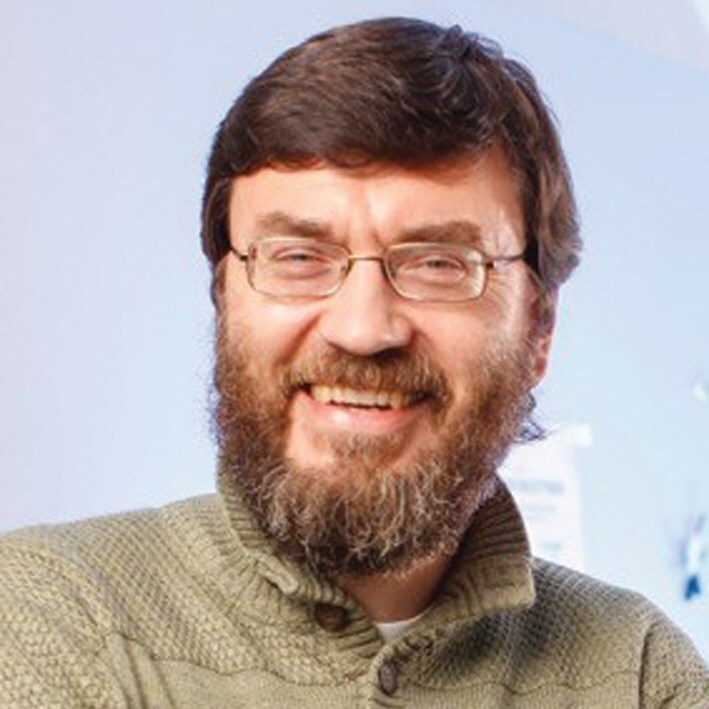


**Andrei Seluanov**

Aging is one of the most fascinating biological phenomena because it affects almost every living organism and biological process. Because aging is such a fundamental biological process, it is important to understand the hierarchical organization of these processes. Which process is a primary driver, which ones are the secondary drivers, and which processes amplify the phenotype? As we already know from our understanding of the hierarchical organization of the cell, most of the cellular processes starts with the DNA. Therefore, I believe, the initial driver of aging has something to do with DNA. Destabilization of the genome (mutations, rearrangements, etc.) and as a result of this destabilization of the epigenome are the likely primary drivers of aging. Boosting genome stability and, most importantly, epigenome stability will most likely lead to life extension.


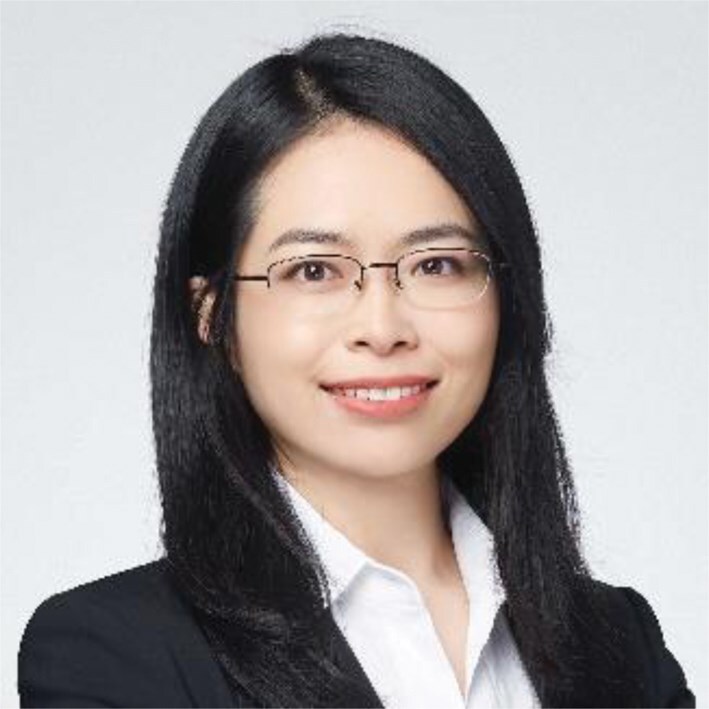


**Moshi Song**

Aging encapsulates the progressive loss of an organism’s adaptive capacity to internal and external stressors. This decline in adaptability manifests as a functional deterioration and an increased vulnerability to diseases, which is governed by a complex interplay between genetic factors and stochastic molecular events. Understanding the intricate interplay between these factors is crucial for developing interventions aimed at promoting healthy aging and extending healthspan.


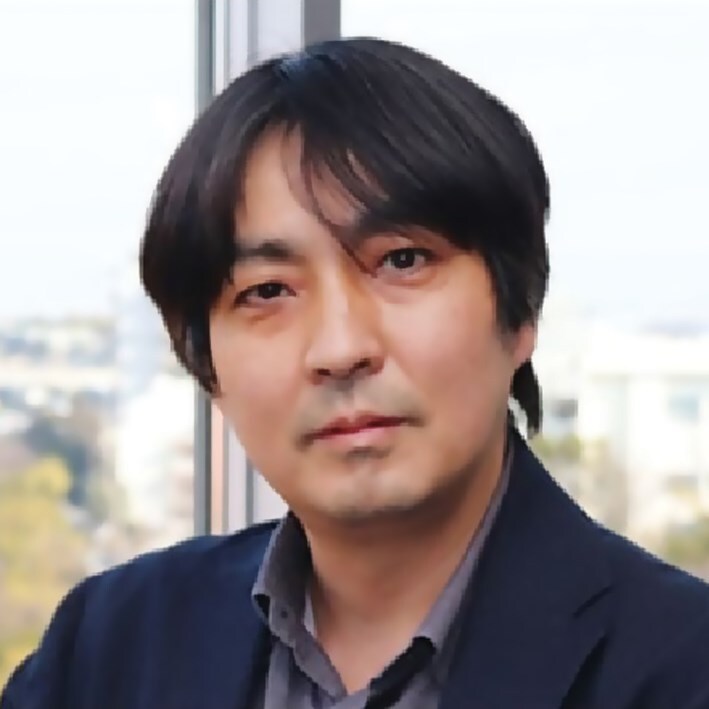


**Keiichiro Suzuki**

Aging is the most complex, multifactorial process in the real world. In other words, the very essence of life itself drives aging. To conquer aging, we must analyze the multifaceted phenomena of life from various angles and fine-tune the many intricate biological processes that sustain us.

